# Acceptance of Public Health Measures During the COVID-19 Pandemic: A Cross-Sectional Study of the Swiss Population’s Beliefs, Attitudes, Trust, and Information-Seeking Behavior

**DOI:** 10.3389/ijph.2023.1605982

**Published:** 2023-06-20

**Authors:** Maddalena Fiordelli, Sara Rubinelli, Nicola Diviani

**Affiliations:** ^1^ Institute of Public Health, Università della Svizzera italiana, Lugano, Switzerland; ^2^ Faculty of Health Sciences and Medicine, University of Lucerne, Lucerne, Switzerland; ^3^ Swiss Paraplegic Research, Nottwil, Switzerland

**Keywords:** trust, infodemic, institutional communication, public health measures, information-seeking behavior

## Abstract

**Objectives:** This study aimed to advance the understanding of the factors associated with population acceptance of public health measures during the COVID-19 pandemic.

**Methods:** In January 2022, we conducted a cross-sectional survey of the Swiss population (*N* = 2,587). Questionnaires were administered through computer-assisted web interviewing. Measures covered included information-seeking behavior, attitudes towards and beliefs about public health measures enacted, and trust in institutions.

**Results:** Television and newspapers were the most used information sources. Those with higher education levels were more likely to use channels from public institutions, newspapers, and television. The most important criterion for reliable information was scientific evidence. Trust was highest for doctors, healthcare workers, universities, research institutes, and public health institutions. Acceptance of public health measures was high overall, and attitudes, beliefs, information-seeking behavior, and trust were positively related to acceptance. Trust in science remained stable, while trust in public health institutions decreased slightly.

**Conclusion:** While nurturing a two-way dialogue with the population, institutions should target communication considering age and culture, improve risk communication, ground messages in scientific evidence, and ensure mass media presence.

## Introduction

On February 11, 2020 a neologism entered vocabularies worldwide: COVID-19. The name the World Health Organization (WHO) announced to designate the disease caused by the novel coronavirus SARS-CoV-2 has now been used for over 3 years [1]. COVID-19 was an unprecedented pandemic, forcing governments to react quickly to limit burdens on the healthcare sector and save lives. Since its onset, governmental institutions have relied on public health policies that require strict adherence by the population: these are physical distancing, self-isolation, or stay-at-home policies.

Reactions to the pandemic by the governments of the world were diverse, and population adherence was in some places suboptimal, even resulting in public protests [2]. Public health and governmental institutions constantly communicated new rules to adapt to the rapidly changing situation. Beyond the informative objective, their communication’s primary and essential goal was persuasive, aimed at generating a consensus and a stable behavioral change for adopting the preventive measures [3–5]. Public institutions played a crucial role in ensuring population adherence, a task that became more complex in the face of an infodemic, defined by WHO as “too much information including false or misleading information in digital and physical environments during a disease outbreak” [6]. Information shapes people’s perception and knowledge about an issue, but it can discourage people from compliance or cause them to take improper actions if the information is difficult to navigate. Public institutions must ensure their presence in multiple communication channels with quality information to grant timely access to critical knowledge [7–9] and find novel ways to leverage trusted sources, as people who believe misinformation and conspiracy theories are less supportive of public health policies [10–13]. The evidence from social sciences could guide the development of better strategies that do not risk falling into ethical pitfalls [5].

Health communication research demonstrated extensively how health perception, trust, and attitudes act as determinants of behavior [14–16]. Although access to information and the ability to evaluate it are essential prerequisites, they are not enough to guarantee adherence to protective behavior. Persuasive communication facilitates health behavior change, and it requires trust in the primary source producing the message, therefore, trust in those defining policies on public health measures [17]. Trust in government communications was associated with adherence to protective behaviors [7, 18–20], both in the general population and in specific subgroups [17, 21]. However, there are no absolute modulators of trust, and little is known about what worked during the COVID-19 pandemic. In the US, the association between socio-demographic characteristics and trust in government, between different information sources use and knowledge about COVID-19, and adherence to social distancing has been proven [7]. In Switzerland, a longitudinal study showed increased corona-specific health literacy levels during the pandemic, with those who reported more trust in different information sources scoring higher [22].

To inform future public health emergency management efforts, monitoring what made institutional decisions acceptable to the public is paramount. The primary aim of our study was to understand the factors associated with the acceptance of governmental public health measures during the COVID-19 pandemic in Switzerland.

## Methods

We conducted a cross-sectional survey of the Swiss population to investigate its information-seeking behavior, attitudes towards and beliefs about the COVID-19 pandemic, the protective measures enforced by public health institutions, and their trust in public institutions. Data were collected in January 2022 through computer-assisted web interviewing (CAWI).

### Questionnaires

A questionnaire was developed in Italian and then translated and back translated into both French and German. Three experienced researchers designed the questions following the conceptual definitions of investigated construct. They checked face validity through constant comparison and with other researchers in the translation phase. The three versions were then pilot tested with a group of students. The estimated completion time was about 10 min. The section dedicated to socio-demographics covered gender, year of birth, nationality, mother tongue, living situation, canton of residence, education, profession, and income. The following section investigated individual health-related information, such as health status, COVID-19 infection status (been infected; not been infected), and COVID-19 vaccination status (vaccinated; not-vaccinated).

#### Information-Seeking Behavior

Information-seeking behavior section measured the criteria used to judge the reliability of the information, the information sources used for COVID-19, the importance of being up-to-date about COVID-19, the perceived ability to look for health information (5-point agreement scale), and an evaluation of the public health institutions’ communications. The latter included a question about the performance of international, national, and local public institutions and a grid evaluating nine criteria for good communication derived by Hyland-Wood et al. [4].

#### Attitudes and Beliefs

Beliefs and attitudes were measured with four sets of questions, all using a 5-point agreement scale. The first set assessed the opinions about the interests (economic, political, public health, and social) guiding public institution decisions during the pandemic. Next was a 14-item matrix assessing the acceptance of the different federal decisions about public health measures presented in chronological order. Reliability analysis proved a high inter-item correlation (0.901) related to the 14 items of the acceptance of public health measures. These were later recoded into a new variable computed as the average of all items. The third set of questions was a three-item scale measuring risk perception, which were highly correlated and then merged into a unique variable after checking reliability with Cronbach’s alpha (0.871). The final set of questions included 17 items describing beliefs about COVID-19 management as reported and discussed by media and institutions during the pandemic. These were later tested separately for reliability. The 12 items related to attitudes on pandemic management had a high Cronbach’s alpha (0.870) and were all retained in a new variable that computed their average. Five items pertaining to beliefs on institutional roles and values were highly reliable if the item “It is important always to ensure that every citizen is free to do as they please” was excluded (0.710). A new variable was then computed with the remaining four items.

#### Trust

Four sets of questions measured trust. The first assessed trust in 15 different information sources, from institutional (e.g., WHO, confederation) to social (e.g., colleagues, family), to be scored on a 5-point scale. Another set of three questions measured the change in trust at three different institutional levels (national, local, and scientific) during the COVID-19 pandemic. The change was measured on a 5-point scale ranging from 1 = My confidence has decreased to 5 = My confidence has increased, and 3 meant no change. The three items of change in trust had a high reliability score (Cronbach’s alpha 0.795) and were grouped into a new variable.

### Data Analysis

All statistical analyses were performed using IBM SPSS Statistics 25 (IBM, Armonk, NY, United States). Frequencies and means were used to describe the sample. Some socio-demographic variables were recoded and later used to compare groups in descriptive analyses. We recoded canton of residence into linguistic regions (Swiss–German, Swiss–French, and Swiss–Italian). Year of birth was recoded into age class (18–39; 40–64; 65+). Education was recoded into level of education: low (no education; mandatory school; general education without scholastic diploma; vocational school or apprenticeship), medium (high school with diploma; higher vocational training; higher vocational school), and high (university or university of applied science; doctorate or habilitation). After checking the assumptions of normality and homogeneity, independent sample *t*-tests, one-way analysis of variations (ANOVAs) or Kruskal-Wallis non-parametric tests were performed to compare the means for information-seeking behavior, attitudes, beliefs, and trust in all groups (see Supplementary Tables S1–S3).

With the acceptance of public health measures as the primary outcome, we performed linear regression analyses with predictor average scores while checking the assumptions of normality, collinearity, and homoscedasticity (see Supplementary Figures S1, S2; Supplementary Table S4). Separate models were calculated for each set of predictors (information-seeking behavior, attitudes and beliefs, and change in trust) to avoid bias arising from overadjustment. All models were adjusted for age, gender, educational level, health status, previous infection, and vaccination status. The resulting standardized coefficients and 95% CIs were reported.

## Results

A total of 2,587 people comprised the final sample. Participants were, on average, 49 years old (*SD*: 16) and were equally distributed in terms of gender (male 49.7%). The majority were Swiss (95%), Swiss–German mother tongue (64%), living with family (70%), and residing in a Swiss–German canton (65%). One in five had been infected at the time of the survey, and the vast majority (85%) were vaccinated (Table 1).

**TABLE 1:** Demographic and descriptive statistics of the study participants. Developing standards for institutional health communication during public health emergencies. Learning from information around COVID-19 pandemic as a case in point, Switzerland, 2020–2022.

Characteristics	Value
Age, mean	49
(SD)	(16)
Age class, % (*n*)	
18–39	33 (856)
40–64	48 (1,243)
65+	19 (488)
Gender, % (*n*)	
Male	49.7 (1,286)
Female	50.1 (1,297)
Citizenship, % (*n*)	
Swiss	95.2 (2,464)
Italian	6.8 (175)
German	2.2 (56)
Portuguese	0.9 (23)
French	2.5 (65)
Spanish	0.8 (20)
Other	4 (107)
Language, % (*n*)	
Swiss German	64.1 (1,658)
German	7.0 (181)
French	25.2 (653)
Tessin dialect	4.6 (118)
Italian	14.6 (377)
Serbian/Croatian	0.9 (23)
Portuguese	1.3 (33)
Spanish	1.3 (33)
English	4.7121)
Other	3.5 (86)
Living arrangement, % (*n*)	
Alone	21.3 (551)
With family	70.2 (1817)
With friends	2.7 (69)
With housemates	5.4 (139)
*Missing*	*0.4(11)*
Linguistic area, % (*n*)	
Swiss German	65 (1,681)
Swiss French	23.5 (603)
Swiss Italian	11.5 (300)
Health status, % (*n*)	
Very bad	0.3 8)
Bad	4.0 (104)
Neither good nor bad	10.4 (269)
Good	51.5 (1,332)
Very good	33.6 (870)
Education, % (*n*)	
Low	46.7 (1,208)
Medium	31.5 (814)
High	21.6 (560)
Occupation, % (*n*)	
Student	6.4 (166)
Employed	59.6 (1,541)
Independent	5.3 (137)
Househusband/Housewife	7 (181)
Retired	19.1 (494)
Unemployed, looking for work	1.4 (35)
Unemployed, not looking for work	0.3 (7)
*Missing*	*1(26)*
Income, % (*n*)	
Below 3,000 CHF	6.3 (163)
3,001–6,000 CHF	23.9 (618)
6,001–9,000 CHF	25 (648)
9,001–12,000 CHF	16.9 (437)
12,001–15,000 CHF	8.3 (214)
More than 15,000 CHF	5.1 (133)
*Missing*	*14.5(374)*
COVID-19 Infection, % (*n*)	
Yes	20.4 (527)
No	74.6 (1930)
*Missing*	*5(130)*
COVID-19 vaccination, % (*n*)	
Vaccinated	84.8 (2,194)
Not vaccinated	12.9 (335)
Don’t know/No answer	2.2 (58)

Source: Cross-sectional survey of Swiss population held in January 2022. Base: 2,587 Swiss residents.

### Information-Seeking Behavior

The information sources used most were television (72.8%) and newspapers (67.4%), followed by public health institutions’ channels (e.g., Federal Office of Public Health website with 55.8%), radio (53.4%), and the internet (51.4%). The least used sources were closed groups on Facebook (0.7%), online forums (0.9%), and public figures (1.7%). Less than 1 in 10 used social media, mainly Facebook (9.5%), WhatsApp (6.9%), and Instagram (4.0%) (Table 2).

**TABLE 2:** Information sources used and criteria used to judge reliable information. Developing standards for institutional health communication during public health emergencies. Learning from information around COVID-19 pandemic as a case in point, Switzerland, 2020–2022.

		Gender		Age			Education			Linguistic region		
	Total	Male	Female	18–39	40–64	65+	Low	Medium	High	Swiss DE	Swiss FR	Swiss IT
Info sources, %												
TV	72.8	72.8	70.5	58.9	78.0	87.4	76.7	72.9	67.9	71.5	75.8	80.5
Newspapers	67.4	7.4	65	63.0	67.3	79.0	64.9	69.1	73.6	70.8	60.1	69.4
Channels from public institutions (e.g., BAG website)	55.8	55.8	54.5	65.0	56.2	41.8	45.5	60.2	74.1	56.9	55.5	54.5
Radio	53.4	53.4	50.9	47.9	55.9	59.7	53.7	54.6	53.9	55.4	48.2	57.2
Internet (search engines)	51.4	51.4	52	56.8	52.9	41.2	47.0	55.4	57.8	51.7	52.9	51.5
Doctors and researchers in the media	43.9	43.9	41.5	40.7	46.8	44.2	36.6	47.8	56.0	47.8	34.4	44.4
Family members	38.5	38.5	41.1	44.3	37.3	33.7	36.9	40.5	40.9	41.3	36.7	30.0
My doctor and other healthcare professionals	35.0	35.0	37.4	33.8	35.7	37.4	36.6	47.8	56.0	33.3	37.9	41.8
Friends	30.8	30.8	31.5	37.2	30.8	21.4	29.3	33.5	31.8	32.7	30.0	24.2
My colleagues	22.0	22	19.7	25.9	25.6	7.4	20.6	24.0	23.5	23.4	22.7	14.8
Politicians	11.4	11.4	11.3	11.5	11.8	10.7	12.0	9.9	12.7	10.6	13.4	12.8
Facebook	9.5	9.5	10.6	12.2	9.2	6.0	11.4	8.8	6.8	7.6	14.3	11.4
WhatsApp	6.9	6.9	6.9	6.3	7.7	6.2	9.0	6.2	3.8	6.7	7.9	6.7
Instagram	4.0	4.0	4.6	9.5	1.6	0.6	3.4	5.4	3.2	3.5	5.2	4.7
Telegram	2.7	2.7	2.5	2.7	3.2	1.1	2.9	2.5	2.5	3.3	2.0	0.7
Scientific divulgers	2.4	2.4	2.1	4.2	1.8	0.8	1.0	2.2	5.6	2.3	2.0	3.4
Twitter	1.7	1.7	1.2	2.9	1.6	—	0.8	2.0	3.4	1.4	2.3	2.0
Public figures (influencers, writers, actors)	1.2	1.2	1.2	1.4	0.9	1.4	1.0	1.5	1.1	1.4	0.8	0.3
Online forums	0.9	0.9	0.8	0.5	0.7	2.1	1.0	0.7	0.9	1.1	0.7	0.3
Facebook closed groups	0.7	0.7	0.8	0.4	0.8	0.8	0.8	0.6	0.5	0.7	0.7	0.3
Criteria for reliable information about COVID-19, %												
It is based on scientific evidence	72.4	74.1	70.7	78.8	72.1	75.6	63.3	81.0	90.3	75.5	74.3	72.7
It helps me to better understand the situation	46.8	45.6	48	50.5	46.2	50.6	44.1	53.2	50.5	50.5	42.6	48.8
It presents more points of view	44.8	42.7	46.9	49.1	47.2	39.5	43.5	50.1	46.9	48.9	45.2	34.6
It is presented by health institutions	39.4	40	38.8	44.0	38.8	40.3	33.6	42.9	52.7	36.6	45.7	54.7
It is based on the anecdotes and the experience of people	27.6	26.7	28.5	29.0	30.3	23.6	32.0	29.7	20.0	31.7	24.7	18.7
It is easy to find and to understand	14.3	14	14.6	12.7	15.6	16.1	14.9	17.0	11.3	17.1	12.6	6.2
It confirms my ideas	8.3	9.6	7.1	6.3	9.2	11	11.4	7.7	4.1	8.4	10.0	6.9
It is shared by many people	3.9	4.4	3.5	3.4	3.9	5.8	6.1	3.0	1.4	3.7	5.5	3.5
It goes against common thinking (mainstream)	2.8	3.3	2.2	3.6	2.9	1.9	3.6	2.0	2.9	2.0	3.8	1.0

Source: Cross-sectional survey of Swiss population held in January 2022. Base: 2,587 Swiss residents.

There were no significant differences between genders, but there were among age classes (Table 2). Television was the first source of information for adults (78.0%) and for older adults (87.4%), while the younger group mainly used channels from public institutions (65%) and newspapers (63%). Educational level also played a role, with those in the highly educated group using channels from public institutions (74.1%), newspapers (73.6%), and television (67.9%) more frequently. Education also distinguished the use of social media, where people with a low educational level used Facebook (11.4%) and WhatsApp (9%) above the general mean.

The most frequently used criterion to judge reliable information about COVID-19 was “it is based on scientific evidence,” which was true across gender, age class, educational level, and linguistic region (Table 2). The order of criteria used was similar across gender, age class, and educational level, with the criterion that scored the lowest being “it goes against common thinking.” Differences were found among linguistic regions, as, aside from the first criterion, the order of the criteria used changed. For those living in the Swiss–German region, other critical criteria (above the general mean) were “it presents more points of view” (48.9%) and “it is based on the anecdotes and the experience of people” (31.7%), while information that was “presented by health institutions” was below the overall mean (36.6%). In the French-speaking region, information that was “presented by health institutions” was a frequently mentioned criterion (45.7% vs. overall mean of 39.4%), and this was even more in the Italian-speaking region (54.7%). In the Swiss–Italian region, information that “presents more points of view” was not among the most relevant criteria (34.6% vs. overall mean of 44.8%).

The perceived ability to find reliable information was overall average (*M*: 3.60, *SD*: 0.9), with no differences for gender but with highly significant differences for age, educational level, and linguistic region (Table 3). This ability increased with age and education, while it was lower in the Swiss–French region. The importance of being updated on the pandemic was also average (*M*: 3.43, *SD*: 1.2), with no difference between groups for gender and educational level, but with significant differences for age class and linguistic region. The importance of being updated on the pandemic increased with age and was higher in the Swiss–Italian region.

**TABLE 3:** Attitudes towards information seeking and trust in information sources. Developing standards for institutional health communication during public health emergencies. Learning from information around COVID-19 pandemic as a case in point, Switzerland, 2020–2022.

		Gender		Age			Education			Linguistic region		
	Total	Male	Female	18–39	40–64	65+	Low	Medium	High	Swiss DE	Swiss FR	Swiss IT
Info seeking												
Ability to find reliable health information sources	3.60 (.09)	3.43 (0.9)	3.43 (0.9)	3.36 (0.9)	3.46 (0.9)	3.52 (0.8)	**3.33(0.9)**	**3.48(0.9)**	**3.60(0.9)**	**3.52(0.9)**	**3.20(0.9)**	**3.43(0.8)**
Importance to be updated on the pandemic	3.43 (1.2)	3.44 (1.2)	3.42 (1.2)	**3.06(1.1)**	**3.47(1.2)**	**3.94(1.1)**	3.37 (1.2)	3.45 (1.2)	3.52 (1.1)	**3.31(1.3)**	**3.60(1.0)**	**3.73(1.0)**
Trust in information sources												
Doctors and healthcare workers	3.88 (0.9)	3.90 (0.9)	3.85 (0.9)	**3.80(0.9)**	**3.84(0.9)**	**4.10(0.8)**	**3.79(0.9)**	**3.91(0.9)**	**4.02(0.8)**	3.91 (0.9)	3.80 (0.9)	3.81 (0.9)
Universities and research institutes	3.82 (1.0)	**3.90(1.0)**	**3.74(1.9)**	**3.81(1.0)**	**3.76(1.0)**	**3.99(0.8)**	**3.60(1.0)**	**3.91(0.9)**	**4.14(0.9)**	3.85 (1.0)	3.76 (1.0)	3.76 (1.0)
Hospitals	3.72 (1.0)	**3.79(1.0)**	**3.65(1.0)**	**3.71(1.0)**	**3.65(1.1)**	**3.92(0.9)**	**3.59(1.0)**	**3.77(1.0)**	**3.90(1.0)**	3.70 (1.0)	3.77 (1.0)	3.71 (1.0)
The Federal Office of Public Health	3.63 (1.1)	3.62 (1.2)	3.64 (1.1)	**3.57(1.2)**	**3.59(1.2)**	**3.84(1.0)**	**3.49(1.2)**	**3.69(1.1)**	**3.84(1.1)**	**3.60(1.2)**	**3.74(1.1)**	**3.58(1.0)**
The chief medical officer	3.62 (1.1)	3.66 (1.1)	3.48 (1.0)	**3.60(1.1)**	**3.55(1.1)**	**3.84(0.9)**	**3.50(1.1)**	**3.65(1.0)**	**3.82(1.0)**	3.59 (1.0)	3.69 (1.1)	3.65 (1.1)
Family members	3.53 (1.0)	3.48 (1.0)	3.58 (1.0)	**3.45(1.0)**	**3.52(1.0)**	**3.68(1.0)**	3.57 (1.0)	3.52 (1.0)	3.43 (1.0)	**3.64(0.9)**	**3.26(1.0)**	**3.40(1.0)**
The Cantonal Department of Health	3.50 (1.0)	3.52 (1.1)	3.48 (1.0)	**3.48(1.0)**	**3.42(1.0)**	**3.71(0.9)**	**3.40(1.1)**	**3.53(1.0)**	**3.69(1.0)**	**3.45(1.0)**	**3.62(1.0)**	**3.57(1.0)**
The Confederation	3.47 (1.1)	3.51 (1.4)	3.43 (1.1)	**3.36(1.1)**	**3.47(1.1)**	**3.67(1.0)**	**3.49(1.2)**	**3.51(1.1)**	**3.68(1.0)**	**3.47(1.1)**	**3.56(1.1)**	**3.27(1.1)**
The Canton	3.34 (1.0)	3.36 (1.0)	3.32 (1.0)	**3.30(1.1)**	**3.30(1.0)**	**3.53(0.9)**	**3.25(1.0)**	**3.38(1.0)**	**3.48(1.0)**	3.30 (1.0)	3.41 (1.0)	3.42 (1.1)
The World Health Organization (WHO)	3.23 (1.1)	3.18 (1.1)	3.29 (1.1)	**3.29(1.1)**	**3.16(1.1)**	**3.32(1.0)**	**3.09(1.1)**	**3.28(1.0)**	**3.47(1.1)**	3.22 (1.1)	3.32 (1.1)	3.12 (1.1)
Friends, acquaintances, colleagues	3.06 (0.9)	3.04 (0.9)	3.08 (0.9)	3.10 (0.9)	3.04 (0.9)	3.04 (0.9)	3.08 (0.9)	3.06 (0.9)	3.03 (0.8)	**3.20(0.9)**	**2.83(0.9)**	**2.73(0.9)**
Journalists	2.50 (1.0)	2.56 (1.0)	2.45 (1.0)	**2.47(1.0)**	**2.46(1.0)**	**2.68(0.9)**	**2.40(1.0)**	**2.51(0.9)**	**2.72(1.0)**	2.52 (1.0)	2.48 (1.0)	2.45 (0.9)
Public figures	2.35 (1.0)	2.35 (1.0)	2.36 (1.0)	**2.15(1.0)**	**2.34(0.9)**	**2.73(1.0)**	**2.45(1.0)**	**2.31(0.9)**	**2.22(0.9)**	**2.40(1.0)**	**2.34(1.0)**	**2.12(0.9)**
Politicians	2.34 (1.0)	**2.28(1.0)**	**2.41(1.0)**	**2.25(1.0)**	**2.34(1.0)**	**2.52(1.0)**	2.34 (1.0)	2.33 (0.9)	2.37 (0.9)	2.34 (1.0)	2.38 (1.0)	2.29 (1.0)
Influencers on social media	1.50 (0.8)	1.50 (0.8)	1.55 (0.7)	**1.55(0.8)**	**1.46(0.7)**	**1.66(0.8)**	**1.63(0.8)**	**1.45(0.7)**	**1.41(0.7)**	1.54 (0.8)	1.51 (0.9)	1.48 (0.8)

Source: Cross-sectional survey of Swiss population held in January 2022. Base: 2,587 Swiss residents. The means in bold indicate the difference among the groups is highly significant (*p* < 0.001). All variables measured on a 5-point agreement Likert scale (1 = Strongly disagree; 5 = Strongly agree).

We also examined the trust attributed to the different information sources (Table 3). At the top of the trusted sources were doctors and healthcare workers (*M*: 3.88, *SD*: 0.9), universities, research institutes (*M*: 3.82, *SD*: 1.0), hospitals (*M*: 3.72, *SD*: 1.0), the Federal Office of Public Health (*M*: 3.63, *SD*: 1.1), and the chief medical officer (*M*: 3.62, *SD*: 1.1). The order of the most trusted sources remained the same across genders, age groups, educational levels, and linguistic regions. Mean differences were consistent and significant for age, with younger age groups being generally more skeptical and those in the older age group having a higher level of trust. Similarly, those with higher education were generally more trusting than those with low education. No significant differences were found for social information sources, such as family members, friends, acquaintances, colleagues, or politicians. Significant mean differences (*p* < 0.001) were sparse for linguistic regions, and they concerned public institutions (Confederation and Cantonal Department of Health) and social information sources. Those living in the Swiss–Italian region trusted the Cantonal Department of Health more than the Swiss–Germans, but less than the Swiss–French. Those residing in the Swiss–Italian region also trusted the Confederation less than the others. The four least trusted sources were influencers on social media (*M*: 1.50, *SD*: 0.8), politicians (*M*: 2.34, *SD*: 1.0), public figures (*M*: 2.35, *SD*: 1.0), and journalists (*M*: 2.50, *SD*: 1.0). Differences in trust in these information sources were registered among age groups and educational levels.

### Attitudes and Beliefs

Scores on all three risk perception items were high (Table 4), with highly significant (*p* < .001) differences across age classes and linguistic regions. The younger group and those residing in the Swiss–French region scored lower.

**TABLE 4:** Attitudes towards pandemic and its management by gender, age, and education. Developing standards for institutional health communication during public health emergencies. Learning from information around COVID-19 pandemic as a case in point, Switzerland, 2020–2022.

		Gender		Age			Education			Linguistic region		
	Total	Male	Female	18–39	40–64	65+	Low	Medium	High	Swiss DE	Swiss FR	Swiss IT
Risk perception												
It is important to put in place preventive measures against COVID-19	4.10 (1.1)	4.10 (1.1)	4.10 (1.1)	**3.90(1.2)**	**4.0(1.1)**	**4.50(0.8)**	**3.99(1.2)**	**4.14(1.1)**	**4.21(1.0)**	**4.08(1.1)**	**3.99(1.1)**	**4.30(1.0)**
COVID-19 is a risk for many people	3.80 (1.2)	3.80 (1.2)	3.80 (1.2)	**3.50(1.2)**	**3.80(1.2)**	**4.40(0.9)**	3.83 (1.2)	3.83 (1.2)	3.85 (1.2)	**3.74(1.2)**	**3.98(1.1)**	**4.11(1.0)**
COVID-19 is a severe illness	3.70 (1.2)	3.70 (1.2)	3.70 (1.2)	**3.40(1.2)**	**3.70(1.3)**	**4.20(0.9)**	3.64 (1.2)	3.7 (1.2)	3.69 (1.1)	**3.70(1.2)**	**3.52(1.2)**	**3.83(1.1)**
Quality of communication during the pandemic												
National Institutions (Bund, BAG)	3.60 (1.1)	**3.50(1.1)**	**3.60(1.0)**	**3.30(1.0)**	**3.60(1.1)**	**3.80(1.0)**	3.60 (1.1)	3.56 (1.1)	3.54 (1.1)	**3.57(1.1)**	**3.73(1.0)**	**3.32(1.1)**
Local Institutions (Canton, health dept.)	3.30 (1.0)	3.20 (1.1)	3.30 (1.0)	**3.10(1.0)**	**3.30(1.0)**	**3.60(0.9)**	3.32 (1.0)	3.32 (1.0)	3.30 (1.0)	**3.21(1.0)**	**3.40(1.0)**	**3.59(1.0)**
International Institutions (WHO)	3.10 (1.0)	**3.0(1.0)**	**3.20(1.0)**	**3.0(1.0)**	**3.0(1.0)**	**3.30(0.9)**	3.12 (1.0)	3.10 (1.0)	3.04 (1.1)	3.09 (1.0)	3.20 (1.0)	2.92 (1.0)
Government decision drivers												
Economic interests	4.0 (0.9)	4.0 (0.9)	4.0 (0.9)	4.0 (0.9)	4.0 (0.9)	4.0 (0.9)	3.99 (1.0)	4.03 (0.9)	3.94 (0.9)	4.01 (0.9)	3.99 (0.9)	3.90 (1.0)
Political interests	3.70 (1.0)	3.60 (1.0)	3.70 (1.0)	3.70 (1.0)	3.70 (1.0)	3.70 (1.0)	3.71 (1.0)	3.74 (1.0)	3.63 (1.0)	**3.79(1.0)**	**3.57(1.0)**	**3.53(1.0)**
Public health interests	3.60 (1.1)	3.70 (1.1)	3.60 (1.1)	**3.50(1.1)**	**3.60(1.1)**	**3.90(0.9)**	**3.56(1.1)**	**3.74(1.0)**	**3.76(1.1)**	3.66 (1.1)	3.69 (1.1)	3.61 (1.0)
Social interests	3.20 (1.0)	3.20 (1.0)	3.20 (1.0)	**3.0(1.0)**	**3.10(1.0)**	**3.40(0.9)**	3.18 (1.0)	3.23 (1.0)	3.12 (1.0)	3.19 (1.0)	3.15 (1.0)	3.24 (1.0)
Public health measures												
The wearing of masks on public transport became mandatory, and entry from high-risk countries was restricted. (July 2020)	4.30 (1.1)	4.30 (1.1)	4.40 (1.0)	**4.10(1.2)**	**4.30(1.1)**	**4.60(0.7)**	4.29 (1.1)	4.36 (1.0)	4.33 (1.1)	**4.33(1.1)**	**4.26(1.0)**	**4.42(0.9)**
Restaurants and bars were reopened outside, as were recreational and sports facilities, and face-to-face classes at universities were allowed again. (April 2021)	4.30 (.9)	**4.20(0.9)**	**4.40(0.8)**	**4.20(1.0)**	**4.40(0.8)**	**4.20(0.8)**	4.27 (0.9)	4.33 (0.8)	4.31 (0.9)	**4.35(0.9)**	**4.21(0.9)**	**4.23(0.9)**
Testing at first symptoms was recommended. (March 2021)	4.20 (1.0)	4.10 (1.0)	4.20 (1.0)	**4.20(1.0)**	**4.10(1.0)**	**4.40(0.8)**	**4.13(1.1)**	**4.27(0.9)**	**4.31(0.9)**	4.24 (1.0)	4.15 (0.9)	4.18 (1.0)
During the stabilization phase, bars and restaurants reopened and the restriction on the number of people at private meetings and events was lifted. (May 2021)	4.2 (1.0)	4.10 (1.0)	4.2 (0.9)	**4.10(1.0)**	**4.20(0.9)**	**4.10(0.9)**	4.14 (1.0)	4.22 (0.9)	4.20 (0.9)	4.18 (1.0)	4.14 (0.9)	4.21 (0.9)
The vaccination campaign has been launched. (January 2021)	4.10 (1.3)	**4.20(1.2)**	**4.0(1.3)**	**3.80(1.3)**	**4.0(1.3)**	**4.60(0.9)**	**3.91(1.4)**	**4.15(1.2)**	**4.32(1.1)**	**4.07(1.3)**	**4.02(1.2)**	**4.25(1.1)**
The COVID certificate has been provided. (June 2021)	4.10 (1.3)	**4.20(1.2)**	**4.0(1.4)**	**3.80(1.4)**	**4.10(1.3)**	**4.60(.9)**	**3.68(1.5)**	**3.96(1.4)**	**4.10(1.3)**	**4.13(1.3)**	**3.97(1.3)**	**4.29(1.2)**
Home office was recommended. (October 2020)	4.10 (1.0)	4.10 (1.0)	4.10 (1.0)	4.10 (1.0)	4.10 (1.0)	4.30 (.9)	**4.08(1.1)**	**4.17(1.0)**	**4.23(1.0)**	**4.19(1.0)**	**4.04(1.0)**	**4.03(1.0)**
The measures were relaxed by opening stores and museums and allowing meetings outside again. (February 2021)	4.10 (.9)	**4.0(1.0)**	**4.20(0.9)**	**4.0(1.0)**	**4.10(1.0)**	**4.40(0.9)**	4.09 (1.0)	4.20 (0.9)	4.15 (0.9)	4.17 (0.9)	4.13 (0.9)	3.98 (1.0)
COVID certificate became obligatory in some public places (restaurants, discotheques) for people over 16 years old. (September 2021)	3.80 (1.5)	**4.0(1.4)**	**3.70(1.5)**	**3.50(1.6)**	**3.80(1.5)**	**4.50(1.1)**	**3.68(1.5)**	**3.96(1.4)**	**4.10(1.3)**	**3.90(1.5)**	**3.65(1.5)**	**4.06(1.3)**
Schools were closed and access to nursing homes was restricted. (March 2020)	3.60 (1.3)	**3.70(1.3)**	**3.50(1.3)**	3.60 (1.2)	3.60 (1.3)	3.80 (1.2)	**3.57(1.3)**	**3.67(1.2)**	**3.75(1.3)**	**3.58(1.3)**	**3.62(1.3)**	**3.99(1.1)**
The requirements for indoor certificates have been tightened - 2G, 2G+ (December 2021)	3.50 (1.6)	3.50 (1.5)	3.40 (1.6)	**3.0(1.6)**	**3.50(1.6)**	**4.30(1.2)**	**3.39(1.6)**	**3.55(1.5)**	**3.65(1.5)**	**3.53(1.6)**	**3.25(1.6)**	**3.80(1.4)**
The SwissCovid tracing app has been made available. (August 2020)	3.50 (1.3)	3.60 (1.3)	3.50 (1.3)	**3.40(1.4)**	**3.50(1.3)**	**4.0(1.1)**	**3.43(1.4)**	**3.58(1.3)**	**3.79(1.3)**	**3.63(1.3)**	**3.44(1.3)**	**3.38(1.4)**
Meetings were limited to a maximum of five people, home offices became mandatory, and stores selling non-essential items were closed. (January 2021)	3.30 (1.4)	3.30 (1.4)	3.30 (1.3)	**3.10(1.4)**	**3.20(1.4)**	**3.70(1.3)**	3.24 (1.4)	3.29 (1.4)	3.46 (1.3)	**3.30(1.4)**	**3.21(1.3)**	**3.46(1.3)**
Restaurants have been closed. (December 2020)	3.10 (1.4)	3.20 (1.4)	3.10 (1.4)	**2.90(1.4)**	**3.10(1.4)**	**3.60(1.3)**	**2.99(1.4)**	**3.17(1.4)**	**3.40(1.3)**	**3.17(1.4)**	**2.97(1.3)**	**3.25(1.4)**

Source: Cross-sectional survey of Swiss population held in January 2022. Base: 2,587 Swiss residents. The means in bold indicate the difference among the groups is highly significant (*p* < 0.001). All variables measured on a 5-point agreement Likert scale (1 = Strongly disagree; 5 = Strongly agree).

The judgment on the quality of communication of federal (*M*: 3.6, *SD*: 1.1), local (*M*: 3.3, *SD*: 1.0), and international institutions (*M*: 3.1, *SD*: 1.0) changed significantly according to age (*p* < .001). The older age group was shown to be more trusting than the younger. Opinions on the drivers of government decisions were similar across groups: economic interests were at the top (*M*: 4.0, *SD*: 0.9), followed by political interests (*M*: 3.7, *SD*: 1.0), public health interests (*M*: 3.6, *SD*: 1.1), and social interest (*M*: 3.2, *SD*: 1.0).

Overall, acceptance of public health measures was high. The decision with the highest agreement was that “the wearing of masks on public transport became mandatory, and entry from high-risk countries was restricted (July 2020)” (*M*: 4.3, *SD*: 1.1), and the one with the lowest agreement was “Restaurants have been closed (December 2020)” (*M*: 3.1, *SD*: 1.4). Highly significant differences (*p* < 0.001) for some of the items were registered for gender, age, educational level, and linguistic region. Acceptance was generally higher among the older age group, while the younger age group showed less approval for measures involving limitations of social contact. Those with a high educational level showed significantly higher acceptance, especially for the vaccination and COVID certificate measures. Residents of the Swiss–French region showed less acceptance than the other linguistic regions, but without a consistent trend in significance.

Attitudes towards and beliefs about pandemic management are represented in Table 5. Those with the highest agreement were: “International scientific community and medical research are critical to understanding how to manage the pandemic” (*M*: 4.0, *SD*: 1.0), “Vaccines are important to limit the pandemic” (*M*: 3.9, *SD*: 1.3), “Vaccination contributes to the solution of the COVID-19 problem” (*M*: 3.8, *SD*: 1.4), “The good of the community is worth more than the freedom of the individual” (*M*: 3.8, *SD*: 1.2), and “Health is more important than economy” (*M*: 3.8, *SD*: 1.1). The elements with the least agreement were: “It is important to always ensure that every citizen is free to do as they please” (*M*: 3.1, *SD*: 1.3), “The institutions are completely transparent” (*M*: 3.1, *SD*: 1.2), “The healthcare system neglects the needs of other (non-COVID) patients” (*M*: 3.1, *SD*: 1.2), “In taking action to prevent COVID-19, the government appropriately considers all the different occupational groups” (*M*: 3.1, *SD*: 1.2), and “The government hears and considers the views of citizens” (*M*: 3.1, *SD*: 1.1).

**TABLE 5:** Attitudes and beliefs towards pandemic management by gender, age, and education. Developing standards for institutional health communication during public health emergencies. Learning from information around COVID-19 pandemic as a case in point, Switzerland, 2020–2022.

		Gender		Age			Education			Linguistic region		
	Total	Male	Female	18–39	40–64	65+	Low	Medium	High	Swiss DE	Swiss FR	Swiss IT
Attitudes and beliefs												
International scientific community and medical research are critical to understanding how to manage the pandemic	4.0 (1.0)	4.0 (1.0)	4.0 (1.0)	**3.90(1.0)**	**3.90(1.0)**	**4.30(0.8)**	**3.86(1.1)**	**4.01(1.0)**	**4.17(1.0)**	**3.49(1.1)**	**3.96(1.0)**	**4.21(0.9)**
Vaccines are important to limit the pandemic	3.90 (1.3)	**4.01(1.3)**	**3.9(1.4)**	**3.70(1.3)**	**3.80(1.4)**	**4.30(1.2)**	**3.79(1.4)**	**3.93(1.3)**	**3.98(1.3)**	**4.05(1.3)**	**3.26(1.3)**	**4.13(1.2)**
Vaccination contributes to the solution of the COVID-19 problem	3.80 (1.4)	**4.0(1.3)**	**3.70(1.4)**	**3.50(1.5)**	**3.80(1.4)**	**4.40(1.0)**	**3.65(1.5)**	**3.93(1.4)**	**4.14(1.2)**	**3.95(1.4)**	**3.47(1.5)**	**3.94(1.3)**
The good of the community is worth more than the freedom of the individual	3.80 (1.2)	3.80 (1.2)	3.80 (1.1)	**3.50(1.2)**	**3.80(1.2)**	**3.80(1.0)**	3.76 (1.2)	3.90 (1.2)	3.83 (1.1)	**3.87(1.2)**	**3.55(1.2)**	**4.01(1.1)**
Health is more important than economy	3.80 (1.1)	3.80 (1.1)	3.90 (1.1)	**3.70(1.2)**	**3.80(1.0)**	**4.20(0.9)**	3.82 (1.1)	3.86 (1.1)	3.96 (1.1)	**3.82(1.0)**	**3.86(1.3)**	**4.13(0.9)**
The COVID certificate is important for the containment of the pandemic	3.70 (1.4)	**3.80(1.3)**	**3.60(1.4)**	**3.40(1.4)**	**3.60(1.4)**	**4.30(1.1)**	**3.56(1.4)**	**3.74(1.3)**	**3.88(1.3)**	3.66 (1.4)	3.69 (1.2)	3.83 (1.3)
The various restrictions on international mobility were appropriate (travel, cross-border commuters)	3.50 (1.3)	3.50 (1.3)	3.50 (1.2)	**3.30(1.3)**	**3.50(1.3)**	**4.0(1.1)**	3.50 (1.3)	3.52 (1.3)	3.54 (1.2)	**3.68(1.2)**	**3.24(1.3)**	**3.51(1.3)**
It is important that institutions decide how the nation should behave	3.50 (1.2)	**3.60(1.2)**	**3.40(1.2)**	**3.30(1.2)**	**3.50(1.2)**	**4.0(1.0)**	**3.42(1.3)**	**3.53(1.2)**	**3.79(1.1)**	**3.43(1.2)**	**3.67(1.4)**	**3.83(1.0)**
The institutions are doing their utmost to solve the COVID-19 problem	3.50 (1.1)	3.50 (1.1)	3.5 (01.1)	**3.20(1.1)**	**3.50(1.1)**	**3.90(1.0)**	3.47 (1.2)	3.55 (1.1)	3.50 (1.1)	**3.57(1.1)**	**3.33(1.1)**	**3.45(1.1)**
Switzerland is doing well in containing the pandemic compared to other countries	3.4 (1.1)	3.40 (1.1)	3.30 (1.1)	**3.0(1.2)**	**3.50(1.1)**	**3.60(1.0)**	3.40 (1.1)	3.38 (1.1)	3.30 (1.2)	**3.33(1.1)**	**3.55(1.2)**	**3.26(1.0)**
Institutions have relied too much on the individual responsibility of citizens	3.30 (1.2)	3.30 (1.3)	3.20 (1.2)	**3.20(1.3)**	**3.20(1.2)**	**3.70(1.1)**	3.30 (1.2)	3.29 (1.2)	3.35 (1.3)	**3.31(1.2)**	**3.08(1.2)**	**3.47(1.1)**
The government hears and considers the views of the experts	3.30 (1.1)	3.30 (1.1)	3.30 (1.1)	**3.10(1.1)**	**3.30(1.1)**	**3.60(1.0)**	3.27 (1.1)	3.39 (1.0)	3.31 (1.1)	**3.45(1.0)**	**2.87(1.2)**	**3.42(1.0)**
It is important to always ensure that every citizen is free to do as they please	3.10 (1.3)	3.0 (1.3)	3.10 (1.3)	3.10 (1.3)	3.10 (1.3)	2.90 (1.4)	**3.18(1.3)**	**3.0(1.3)**	**2.91(1.3)**	**2.93(1.3)**	**3.59(1.3)**	**3.07(1.3)**
The institutions are completely transparent	3.10 (1.2)	3.10 (1.2)	3.0 (1.2)	**2.90(1.2)**	**3.0(1.2)**	**3.40(1.0)**	**3.04(1.2)**	**3.05(1.2)**	**3.21(1.2)**	**2.79(1.1)**	**4.01(1.1)**	**2.81(1.1)**
The healthcare system neglects the needs of other (non-covid) patients	3.10 (1.2)	3.10 (1.2)	3.10 (1.2)	3.10 (1.2)	3.20 (1.2)	3.10 (1.1)	3.17 (1.2)	3.10 (1.2)	3.18 (1.1)	**3.22(1.2)**	**2.88(1.2)**	**3.31(1.1)**
In taking action to prevent COVID-19, the government appropriately considers all the different occupational groups	3.10 (1.2)	**3.20(1.2)**	**3.0(1.1)**	**2.80(1.2)**	**3.0(1.1)**	**3.60(1.0)**	3.08 (1.2)	3.12 (1.2)	3.10 (1.2)	**3.02(1.1)**	**3.43(1.2)**	**2.91(1.1)**
The government hears and considers the views of citizens	3.10 (1.1)	3.20 (1.1)	3.10 (1.1)	**3.0(1.1)**	**3.10(1.1)**	**3.50(1.0)**	**3.06(1.2)**	**3.21(1.0)**	**3.31(1.1)**	**3.08(1.1)**	**3.55(1.1)**	**2.88(1.1)**

Source: Cross-sectional survey of Swiss population held in January 2022. Base: 2,587 Swiss residents. The means in bold indicate the difference among the groups is highly significant (*p* < 0.001). All variables measured on a 5-point agreement Likert scale (1 = Strongly disagree; 5 = Strongly agree).

Significant differences were consistent among age groups (*p* < 0.001), with older adults showing stronger attitudes and beliefs. Significant differences were not evident for educational levels, but the highly educated showed stronger attitudes and beliefs related to those items involving scientific grounding of pandemic management and the government’s decisive role. An exception to this was the item “It is important always to ensure that citizens are free to do as they please,” with lower educational levels holding this belief more strongly. Consistent mean differences were found for linguistic regions; the main gaps in the agreement were between Swiss–French and the other two linguistic regions.

### Trust

Change in trust during the pandemic for science was on average 2.99 (scale 1-decreased to 5-increased, SD:0.8), close to the central point, which stands for no change in trust. Change in trust for cantonal public health institutions was on average 2.64 (SD: 0.8), and for federal public health institutions 2.59 (SD: 1.0). No differences for gender and linguistic regions were found, while significant differences for age groups and educational levels were registered for all three variables. The younger age group trusted science slightly more, while the older age group trusted both cantonal and federal institutions more than the younger age group. Those with higher educational levels were shown to be more trustful.

Correlations among the three variables related to the change in trust during the pandemic were moderate to high and all significant. The correlation between change in trust in federal public health institutions and cantonal health institutions was high and significant (*R* = 0.720**).

#### Regression

The results from the multiple linear regression analyses predicting the acceptance of public health measures during the COVID-19 pandemic are summarized in Table 6. The primary constructs investigated in this paper, attitudes towards and beliefs about the pandemic and information-seeking behavior and trust were all significantly positively related to the acceptance of public health measures.

**TABLE 6:** R2, (Δ) R2, Standardized coefficients, and confidence intervals from separate linear regression analyses of acceptance of public health measures during COVID-19 pandemic and attitudes, beliefs, information seeking behavior, and trust. Developing standards for institutional health communication during public health emergencies. Learning from information around COVID-19 pandemic as a case in point, Switzerland, 2020–2022.

Predictors	Outcome (scale 1–5)			
	Scale	R2	(Δ)R2	B (95% CI)
Attitudes and Beliefs				
Attitudes and beliefs towards pandemic management		0.720	0.377	
Attitudes towards institutions’ pandemic management	(1–5)			0.542**(0.498–0.586)
Beliefs about institutional role and values	(1–5)			0.302**(0.265–0.339)
Risk perception	(1–5)	0.586	0.263	0.473**(0.448–0.497)
Judgment on institutional communication	(1–5)	0.533	0.199	0.444**(0.414–0.474)
Appreciation of institutional communication		0.454	0.124	
National institutions (e.g., BAG)				0.171**(0.136–0.206)
International institutions (e.g., WHO)				0.108**(0.075–0.141)
Local institutions (e.g.,,Cantons)				0.056*(0.021–0.091)
Opinion on drivers of public health institutions decision		0.441	0.120	
Public health interests	(1–5)			0.200**(0.171–0.228)
Economic interests	(1–5)			0.097**(0.068–0.127)
Social interests	(1–5)			0.080**(0.052–0.109)
Political interests	(1–5)			−0.065**(−0.092 to −0.037)
Information seeking behavior				
Trust in information sources		0.579	0.250	
Institutional sources	(1–5)			0.554**(−0.513–0.596)
Social relations	(1–5)			0.008 (−0.022–0.037)
Digital sources	(1–5)			−0.036 (−0.078–0.007)
Opinion makers	(1–5)			−0.057 (−0.127–0.012)
Information seeking		0.397	0.077	
Importance of being up to date	(1–5)			0.156**(0.134–0.179)
Ability to look for health information	(1–5)			0.127**(0.098–0.156)
Trust				
Change in institutional trust	(1–5)	0.445	0.123	0.371**(0.338–0.404)

Source: Cross-sectional survey of Swiss population held in January 2022. Base: 2,587 Swiss residents. Models adjusted for age, gender, educational level, linguistic region, health status, vaccination status, and previous infection with Sars-CoV-2.

*p < 0.05, **p < 0.001.

## Discussion

This paper describes the Swiss population’s information-seeking behavior, attitudes towards and beliefs about the pandemic and its management by public institutions as well as trust in the latter. Additionally, it shows factors associated with the acceptance of public health measures during the COVID-19 pandemic in Switzerland. Swiss residents seemed to accept the different public health measures enacted over almost 2 years of the pandemic, and some factors could explain a more substantial acceptance. Some findings are worth noting, which have clear implications for public institutions in managing a public health crisis. We will present them by dividing the implications related to the content (what) and the mode (how) of institutional communication.

When it comes to the content of communication, an important result of our study concerns the impact of risk perception on the acceptance of preventive measures. Risk perception strongly predicts acceptance and adherence, as it is theoretically supported by protection motivation theory and empirically supported by recent evidence [23, 24]. However, the modulators of risk perception are diverse (e.g., from personality to experience), and risk communication can hardly account for all these differences [25]. Moreover, risk perception is essential for reducing misperception and as a barrier to misinformation and disinformation. A higher level of risk perception can activate the systematic processing of information [26], thus protecting individuals from bias in reasoning. Risk communication is a difficult task, and over the long term, it is difficult to maintain a high level of risk perception in a population. As suggested by the JCIH Editorial Team [27], a community-based approach to risk communication could be a viable and effective solution. This would facilitate the accomplishment of the four interrelated tasks necessary to fulfill science communications mission [28].

Trust was also shown to impact acceptance of preventive measures, in line with the initial assumption and existing evidence [17]. Trust in institutions decreased on average, while trust in science remained the same. This was found to be true across the population, but the younger age group and the lower-educated group were even less trustful. The criterion used more widely across the population (gender, age class, and educational level) to judge a reliable piece of information about the pandemic was that it “is based on scientific evidence.” This has implications for what to communicate: messages that are communicated should be strongly backed up by scientific arguments, and those arguments should be embedded into public institution messages [29].

A second significant set of implications concerns how to communicate. An essential result of this study relates to communication channels, meaning the information sources used during the pandemic. De Gani et al. [22] had already showed that traditional media were the most frequently used by the public at our latitude during the different waves of their longitudinal study. Our study confirms this and adds that, even if there are differences in the preferences of single sources among age groups, the trend is similar across ages. During the pandemic, public institutions communicated using many communication channels, and some even started to be (more) present on social media. As this takes a considerable concerted effort, especially where the teams lack communication experts, it is essential to know that if using new channels is even a viable option for public institutions, when it comes to public health crises, the main channels to concentrate on remain traditional media.

Our findings provide evidence that how receivers’ characteristics, that is, age, educational level, and linguistic region, impact information-seeking, the perception of institutional roles, and the opinions on the drivers of public health decisions and pandemic management. We found that the ability to look for health information increases with age and education, and it is lower in the French-speaking region of Switzerland. The importance of being up-to-date increases with age and is higher in the Swiss–Italian region. Older adults are less critical of the quality of institutional communication during the pandemic than those who have stronger attitudes and beliefs regarding the institutional role and the management of the pandemic. Those with higher education also showed stronger attitudes and beliefs, especially when it came to items with a scientific grounding or the decisive role of institutions in managing the pandemic. This means that if the channel effort can be contained, the design of messages should complexify. Institutional messages must be designed to target different age groups, literacy, and educational levels, and decisions at the federal level should be communicated in a culturally sensitive way. The Swiss health system is very fragmented [30], and this brings extra challenges and opportunities. Still, it notably underscores the ever-existing necessity of accounting for cultural differences in Switzerland. After the first wave of the pandemic, we assisted in a shift from cantonal to federal responsibilities in managing the pandemic. While this was the right decision given the size of the issue, a federal–cantonal collaboration must be strengthened to culturally translate decisions locally.

The differences among subgroups of the population highlight some groups more at risk of dis- and misinformation. This is the case for the Swiss–French, who generally have a lower risk perception, a lower ability to look for health information, and a lower acceptance of public health measures. Previous evidence highlighted cultural differences’ role in health in Switzerland and suggested communication strategies that may account for this [31, 32]. Another critical subgroup is that of younger citizens, who showed less acceptance than other age groups for preventive measures, especially for those involving limitations of social contacts. This is in line with current evidence on the impact of the pandemic on younger populations, and it is a plausible explanation for the study findings showing a lack of adherence in the younger population [33]. Communication with the younger population is a widespread problem, as younger subgroups of the population have often been neglected in receiving governmental communication or need to be more adequately engaged [4].

Last, but not least, if critical subgroups of the population need to be explicitly targeted, a clear majority also needs to be supported. A virtuous circle is detectable that links trust in institutions, beliefs about their role, and, ultimately, acceptance of institutional public health measures, and that is what most Swiss citizens have in common. This virtuous circle should be nurtured and never be taken for granted by adopting a public health approach capable of establishing a two-way dialogue with society, which originates from social listening [34].

### Limitations

We have to acknowledge some limitations of this study. Selection bias is the first one, which is something shared with many research papers. While the sample was well distributed among educational levels, the oldest old were underrepresented, and there was a discrepancy between vaccinated individuals (84%) and actual reality (70%) [35]. The survey was administered online, and even though internet penetration rates are high in Switzerland, this option excluded those unfamiliar with it.

A second limitation refers to our operationalization of trust. We recognize that trust is a broad and contested field and that a complex measurement could better capture the construct multifaceted nature. However, trust was not our solely focus but one of the constructs we considered and checked as determinants of adherence behavior.

The cross-sectional design of this study did not allow for observing changes in acceptance. Although looking for changes was not the study’s aim, comparing acceptance to measures at different time points would still be worthwhile. Though we believe that the timing of the data collection was highly informative, being that the pandemic and restrictive measures were still very present in everyday life in Switzerland and at the top of the news for almost 2 years. Also, the collection was done right before other global events took over the media and agenda setting.

### Conclusion

Our study suggests areas of improvement in institutional communication, both in terms of resources that can be saved and the additional effort needed. Swiss institutions were able to count on a good acceptance of public health decisions made during the pandemic across the population. However, different socio-demographic and cognitive factors can explain lower acceptance levels. Thus, they need to be accounted for in targeted communication with the population. The unprecedented crisis of the recent pandemic presented an opportunity to learn about future emergencies and to push to establish an enduring dialogue with the entire population.
