# Supplementary material for: Acceptance of Public Health Measures During the COVID-19 Pandemic: A Cross-Sectional Study of the Swiss Population’s Beliefs, Attitudes, Trust, and Information-Seeking Behavior
Source: Int J Public Health. 2023 Jun 20;68:1605982. doi: 10.3389/ijph.2023.1605982 (PMC10318558; doi:10.3389/ijph.2023.1605982)
Supplement: Supplementary file 1 [file DataSheet2.docx]

**Supplementary table 1. Assumptions testing for means comparisons on attitudes towards information seeking and trust in information sources. Switzerland, 2022**

|  | **Total** | | **Gender** | **Age** | **Education** | **Linguistic region** |
| --- | --- | --- | --- | --- | --- | --- |
|  | **Skewness** | **Kurtosis** | **Levene’s test Sig.** | **Levene’s test Sig.** | **Levene’s test Sig.** | **Levene’s test Sig.** |
| **Info seeking** |  |  |  |  |  |  |
| Ability to find reliable health information sources | -.424 | -.300 | .876 | .001 | .78 | .083 |
| Importance to be updated on the pandemic | -.430 | -.834 | .944 | .000 | .06 | .000 |
| **Trust in information sources** |  |  |  |  |  |  |
| Doctors and healthcare workers | -.804 | .675 | .050 | .000 | .000 | .076 |
| Universities and research institutes | -.763 | .348 | .060 | .000 | .000 | .874 |
| Hospitals | -.785 | .274 | .392 | .000 | .000 | .866 |
| The Federal Office of Public Health | -.749 | -.172 | .110 | .000 | .000 | .001 |
| The chief medical officer | -.756 | .092 | .859 | .000 | .001 | .104 |
| Family members | -.353 | -.263 | .997 | .607 | .41 | .417 |
| The Cantonal Department of Health | -.606 | -.046 | .135 | .000 | .006 | .703 |
| The Confederation | -.633 | -.313 | .937 | .000 | .000 | .477 |
| The Canton | -.508 | -.172 | .009 | .047 | .55 | .115 |
| The World Health Organization (WHO) | -.382 | -.440 | .948 | .000 | .725 | .678 |
| Friends, acquaintances, colleagues | -.132 | -.079 | .780 | .041 | .011 | .305 |
| Journalists | .010 | -.779 | .052 | .000 | .000 | .046 |
| Public figures | .205 | -.636 | .486 | .533 | .000 | .726 |
| Politicians | .220 | -.649 | .171 | .653 | .000 | .317 |
| Influencers on social media | 1.589 | 2.443 | .894 | .000 | .000 | .637 |

**Supplementary table 2. Assumptions testing for means comparisons on attitudes pandemic and its management by gender, age, and education. Switzerland, 2022**

|  | **Gender** | | **Gender** | **Age** | **Education** | **Linguistic region** |
| --- | --- | --- | --- | --- | --- | --- |
|  | **Skewness** | **Kurtosis** | **Levene’s test Sig.** | **Levene’s test Sig.** | **Levene’s test Sig.** | **Levene’s test Sig.** |
| **Risk perception** |  |  |  |  |  |  |
| It is important to put in place preventive measures against COVID-19 | -1.178 | .525 | .283 | .000 | .003 | .097 |
| COVID-19 is a risk for many people | -.778 | -.389 | .167 | .000 | .08 | .000 |
| COVID-19 is a severe illness | -.583 | -.528 | .636 | .000 | .016 | .054 |
| **Quality of communication during the pandemic** |  |  |  |  |  |  |
| National Institutions (Bund, BAG) | -.706 | -.048 | .000 | .000 | .892 | .217 |
| Local Institutions (Canton, health dept.) | -.475 | -.270 | .002 | .135 | .177 | .465 |
| International Institutions (WHO) | -.328 | -.366 | .376 | .916 | .108 | .314 |
| **Government decision drivers** |  |  |  |  |  |  |
| Economic interests | -.947 | .821 | .777 | .038 | .001 | .016 |
| Political interests | -.540 | -.186 | .098 | .492 | .395 | .000 |
| Public health interests | -.726 | .002 | .642 | .000 | .168 | .218 |
| Social interests | -.336 | -.394 | .827 | .258 | .274 | .983 |
| **Public health measures** |  |  |  |  |  |  |
| The wearing of masks on public transport became mandatory, and entry from high-risk countries was restricted. (July 2020) | -1.740 | 2.304 | .003 | .000 | .075 | .007 |
| Restaurants and bars were reopened outside, as were recreational and sports facilities, and face-to-face classes at universities were allowed again. (April 2021) | -1.431 | 2.211 | .003 | .000 | .107 | .861 |
| Testing at first symptoms was recommended. (March 2021) | -1.366 | 1.531 | .479 | .002 | .003 | .124 |
| During the stabilization phase, bars and restaurants reopened and the restriction on the number of people at private meetings and events was lifted. (May 2021) | -1.217 | 1.182 | .267 | .001 | .562 | .019 |
| The vaccination campaign has been launched. (January 2021) | -1.275 | .414 | .000 | .000 | .000 | .001 |
| The COVID certificate has been provided. (June 2021) | -1.357 | .533 | .001 | .000 | .000 | .001 |
| Home office was recommended. (October 2020) | -1.241 | 1.096 | .744 | .008 | .087 | .801 |
| The measures were relaxed by opening stores and museums and allowing meetings outside again. (February 2021) | -1.149 | 1.089 | .047 | .083 | .377 | .891 |
| COVID certificate became obligatory in some public places (restaurants, discotheques) for people over 16 years old. (September 2021) | -.986 | -.555 | .000 | .000 | .000 | .000 |
| Schools were closed and access to nursing homes was restricted. (March 2020) | -.681 | -.661 | .001 | .001 | .009 | .000 |
| The requirements for indoor certificates have been tightened - 2G, 2G+ (December 2021). | -.551 | -1.270 | .001 | .000 | .002 | .000 |
| The SwissCovid tracing app has been made available. (August 2020) | -.570 | -.853 | .519 | .000 | .005 | .445 |
| Meetings were limited to a maximum of five people, home offices became mandatory, and stores selling non-essential items were closed. (January 2021) | -.328 | -1.098 | .333 | .05 | .793 | .002 |
| Restaurants have been closed. (December 2020) | -.171 | 1.096 | .534 | .005 | .369 | .000 |

**Supplementary table 3. Assumptions testing for means comparisons on attitudes and beliefs towards pandemic management by gender, age, and education. Switzerland, 2022**

|  | **Total** | | **Gender** | **Age** | **Education** | **Linguistic region** |
| --- | --- | --- | --- | --- | --- | --- |
|  | **Skewness** | **Kurtosis** | **Levene’s test Sig.** | **Levene’s test Sig.** | **Levene’s test Sig.** | **Levene’s test Sig.** |
| **Attitudes and beliefs** |  |  |  |  |  |  |
| International scientific community and medical research are critical to understanding how to manage the pandemic | -.926 | .322 | .993 | .000 | .000 | .096 |
| Vaccines are important to limit the pandemic | -.912 | -.48 | .000 | .000 | .027 | .089 |
| Vaccination contributes to the solution of the COVID-19 problem | -.943 | -.506 | .000 | .000 | .000 | .000 |
| The good of the community is worth more than the freedom of the individual | -.839 | -.202 | .005 | .000 | .071 | .04 |
| Health is more important than economy | -.81 | -.007 | .089 | .000 | .022 | .000 |
| The COVID certificate is important for the containment of the pandemic | -.753 | -.7 | .000 | .000 | .000 | .000 |
| The various restrictions on international mobility were appropriate (travel, cross-border commuters). | -.556 | -.723 | .210 | .000 | .614 | .086 |
| It is important that institutions decide how the nation should behave | -.616 | -.515 | .999 | .000 | .000 | .000 |
| The institutions are doing their utmost to solve the COVID-19 problem. | -.55 | -.405 | .661 | .000 | .021 | .319 |
| Switzerland is doing well in containing the pandemic compared to other countries. | -.412 | -.511 | .368 | .054 | .076 | .000 |
| Institutions have relied too much on the individual responsibility of citizens. | -.354 | -.852 | .029 | .000 | .204 | .048 |
| The government hears and considers the views of the experts. | -.453 | -.454 | .904 | .005 | .225 | .000 |
| It is important to always ensure that every citizen is free to do as they please | -.013 | -1.153 | .341 | .003 | .16 | .577 |
| The institutions are completely transparent | -.14 | -.845 | .070 | .093 | .005 | .035 |
| The health care system neglects the needs of other (non-covid) patients. | -.212 | .801 | .464 | .096 | .023 | .011 |
| In taking action to prevent COVID-19, the government appropriately considers all the different occupational groups. | -.259 | -.74 | .014 | .051 | .247 | .005 |
| The government hears and considers the views of citizens. | -.345 | -.57 | .029 | .111 | .014 | .444 |

**Supplementary table 4. Assumptions testing for multiple linear regression. Switzerland, 2022**

| **Pedictors** | **Collinearity** |
| --- | --- |
|  | **VIF** |
| **1 Attitudes and Beliefs** |  |
| **Attitudes & beliefs towards pandemic management** |  |
| Attitudes towards institutions’ pandemic management | 5.633 |
| Beliefs about institutional role and values | 3.126 |
| **Risk perception** | 2.824 |
| **Judgment on institutional communication** | 5.308 |
| **Appreciation of institutional communication** |  |
| National institutions (e.g. BAG) | 3.042 |
| International institutions (e.g. WHO) | 2.000 |
| Local institutions (e.g Cantons) | 2.135 |
| **Opinion on drivers of public health institutions decision** |  |
| Public health interests | 2.055 |
| Economic interests | 1.288 |
| Social interests | 1.491 |
| Political interests | 1.366 |
| **2 Information seeking behavior** |  |
| **Trust in information sources** |  |
| Institutional sources | 5.661 |
| Social relations | 1.278 |
| Digital sources | 1.494 |
| Opinion makers | 2.607 |
| **Information seeking** |  |
| Importance of being up to date | 1.297 |
| Ability to look for health information | 1.232 |
| **3 Trust** |  |
| **Change in institutional trust** | 2.470 |
|  |  |

**Supplementary Figure 1. Regression Normal P-P Plot. Switzerland, 2022**

**
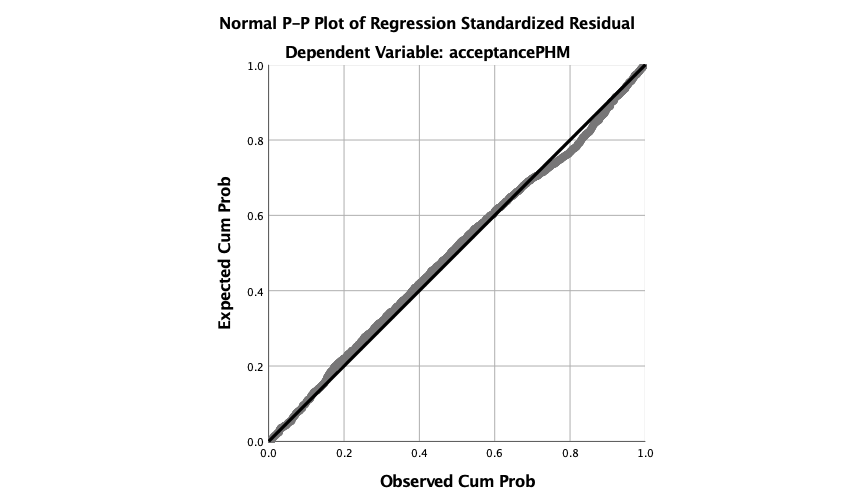
**

**Supplementary Figure 1. Regression Scatterplot. Switzerland, 2022**

**
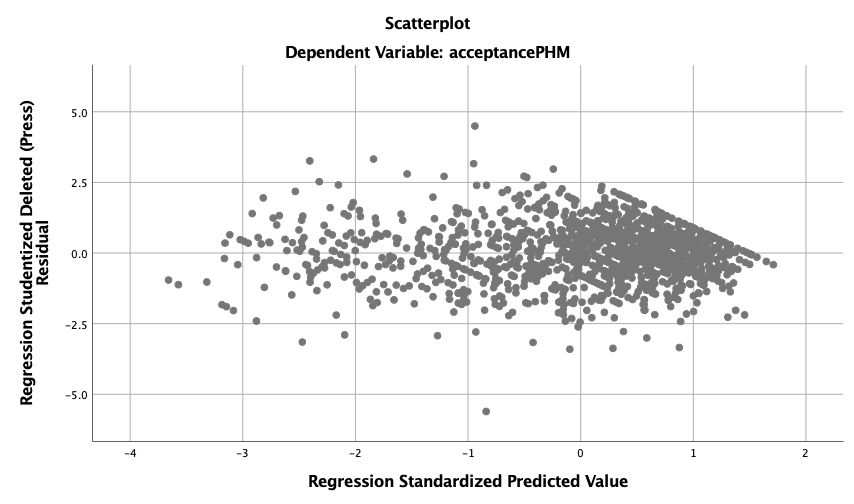
**
