# Supplementary material for: Acceptance of Public Health Measures During the COVID-19 Pandemic: A Cross-Sectional Study of the Swiss Population’s Beliefs, Attitudes, Trust, and Information-Seeking Behavior
Source: Int J Public Health. 2023 Jun 20;68:1605982. doi: 10.3389/ijph.2023.1605982 (PMC10318558; doi:10.3389/ijph.2023.1605982)
Supplement: Supplementary file 2 [file DataSheet1.docx]

**Supplementary material - Original survey questionnaire in Italian**

Buongiorno e grazie per aver accettato di prendere parte a questa indagine sulla ricerca di informazioni e gli atteggiamenti rispetto alle decisioni istituzionali durante la pandemia da COVID-19. Il seguente sondaggio comprende 28 domande e la compilazione dura circa 10 minuti.

Per rendere più scorrevole la lettura, si rinuncia a utilizzare contemporaneamente la forma maschile e femminile. I riferimenti a persone sono da intendersi indicativi di entrambi i generi.

Lo studio è condotto dall’Università di Lucerna e finanziato dal Fondo Nazionale Svizzero per la Ricerca Scientifica.

**Genere**

- Maschile
- Femminile
- Altro

**Data di nascita**________

**Nazionalità** *(più risposte possibili)*

- Svizzera
- Tedesca
- Francese
- Italiana
- Altra(e): _____

**Lingua madre**

- Italiana
- Svizzera-tedesca
- Tedesca
- Francese
- Altra(e):______________

**Lei vive**

- Da solo
- Con i familiari
- Con amici
- Con dei coinquilini

**Cantone di residenza**

| - AG Argovia - AI Appenzello interno - AR Appenzello esterno - BE Berna - BS Basilea città - BL Basilea campagna - FR Friborgo - GE Ginevra - GL Glarona - GR Grigioni - JU Jura - LU Lucerna - NE Neuchâtel | - NW Nidwaldo - OW Obwaldo - SG S. Gallo - SH Sciaffusa - SO Soletta - SZ Svitto - TG Turgovia - TI Ticino - UR Uri - VD Vaud - VS Vallese - ZG Zugo - ZH Zurigo |
| --- | --- |

**Come giudica attualmente il suo stato di salute?**

- 1- Pessimo
- 2
- 3
- 4
- 5 – Molto buono

**Qual è il livello di formazione più alto che ha conseguito? Formazione conseguita significa aver ottenuto un attestato o un diploma.**

- Nessuna (non ha frequentato o non ha portato a termine la scuola dell'obbligo)
- Scuola dell'obbligo
- Formazione generale senza maturità (scuola media di diploma, di cultura generale, d'amministrazione)
- Formazione professionale di base o tirocinio professionale (scuola professionale a tempo pieno, scuola superiore di commercio, scuola di arti e mestieri o formazione equivalente)
- Maturità (liceale, professionale o specializzata)
- Formazione professionale superiore (con attestato professionale federale, diploma federale o maestria)
- Scuola professionale superiore (tecnica ST, economia HF) o scuola tecnica superiore STS, SSQUEA, SSAA, HFS
- Scuola universitaria professionale o università (bachelor, master, licenza, diploma, esame di stato, postgrado)
- Dottorato o abilitazione

**Qual è la sua occupazione principale?**

- Studente
- Lavoratore dipendente
- Lavoratore indipendente
- Pensionato
- Disoccupato in cerca di occupazione
- Disoccupato non in cerca di occupazione

**Qual è il redditto lordo mensile del suo nucleo familiare?**

- <3000 CHF
- 3000 – 6000 CHF
- 9000 – 12000 CHF
- 12000 – 15000 CHF
- 15000 – 18000 CHF
- 18000 – 21000 CHF
- > 21000 CHF
- Nessuna risposta

**Ritiene che sia facile o difficile per lei trovare informazioni affidabili che riguardano la salute?**

- 1 – Molto difficile
- 2
- 3
- 4
- 5 – Molto facile
- Nessuna risposta

**In relazione al COVID-19, ai trattamentei e ai vaccini, quali criteri utilizza per valutare l’affidabilità dell’informazione?** *(più risposte possibili)*

Trovo affidabile l’informazione che…

- - conferma le mie idee
  - mi aiuta a capire meglio la situazione
  - va contro il pensiero comune (*mainstream*)
  - presenta più punti di vista
  - è basata su evidenze scientifiche
  - è presentata dalle istituzioni sanitarie
  - si basa sull’esperienza e il vissuto delle persone
  - è facile da trovare e da capire
  - è condivisa da tanta gente

**Lei ha contratto il COVID-19?**

- - Sì
  - No
  - Non so
  - Nessuna risposta

**Quali fonti ha utilizzato o da quali ha ricevuto informazioni riguardo al COVID-19 durante l’ultimo anno e mezzo?** *(più risposte possibili)*

- Facebook generale
- Facebook – Gruppi chiusi. Per favore indicare quali:____________________________
- Internet (Google etc.)
- Twitter
- Instagram
- Telegram
- WhatsApp
- Forum online. Per favore indicare quali:_______________________________________________
- Giornali
- Radio
- TV
- I canali delle istituzioni pubbliche (es. sito web dell’UFSP)
- Il mio medico e/o altri professionisti sanitari
- I politici
- I medici e/o i ricercatori attraverso i media
- Personaggi pubblici (influencer, scrittori, attori etc.). Per favore indicare quali____________________________________________________
- I divulgatori scientifici. Per favore indicare quali:__________________________________________________
- I miei amici
- I miei familiari
- I miei colleghi
- Altro (Specificare):_________________________________________

**Per me è importante essere sempre aggiornato sull’evoluzione della pandemia.**

- 1 – Fortemente in disaccordo
- 2
- 3
- 4
- 5 – Fortemente d’accordo

**Come hanno comunicato le istituzioni durante la pandemia di COVID-19? Ci riferiremo dapprima alle istituzioni locali, poi nazionali e internazionali.**

Credo che le seguenti istituzioni durante la pandemia abbiano comunicato più o meno bene:

|  | 1- per niente bene | 2 | 3 | 4 | 5 – molto bene |
| --- | --- | --- | --- | --- | --- |
| Istituzioni locali (Cantone, DSS) |  |  |  |  |  |
| Istituzioni nazionali (Confederazione, BAG) |  |  |  |  |  |
| Istituzioni internazionali (OMS) |  |  |  |  |  |

**Come hanno comunicato le istituzioni pubbliche durante il COVID-19? Valuti secondo i seguenti criteri attribuendo un punteggio per ogni criterio elencato.**

Credo che la comunicazione delle istituzioni abbia dimostrato…

|  | 1- per niente | 2 | 3 | 4 | 5 – moltissimo |
| --- | --- | --- | --- | --- | --- |
| Trasparenza |  |  |  |  |  |
| Chiarezza |  |  |  |  |  |
| Qualità |  |  |  |  |  |
| Rilevanza |  |  |  |  |  |
| Onestà |  |  |  |  |  |
| Coerenza |  |  |  |  |  |
| Sensbilità |  |  |  |  |  |
| Capacità di ascolto |  |  |  |  |  |
| Immedesimazione nei cittadini |  |  |  |  |  |

**Quanta fiducia ha nelle seguenti fonti di informazione?**

*Indichi per ciascuna fonte il suo livello di fiducia.*

|  | 1- non mi fido affatto | 2 | 3 | 4 | 5 – mi fido moltissimo |
| --- | --- | --- | --- | --- | --- |
| La Confederazione |  |  |  |  |  |
| L’Ufficio Federale di Sanità Pubblica |  |  |  |  |  |
| Il Cantone (dove risiede) |  |  |  |  |  |
| Il dipartimento di sanità e socialità del suo cantone |  |  |  |  |  |
| Il medico cantonale |  |  |  |  |  |
| Gli ospedali |  |  |  |  |  |
| Le università e gli istituti di ricerca |  |  |  |  |  |
| L’Organizzazione Mondiale della Sanità (OMS) |  |  |  |  |  |
| I giornalisti |  |  |  |  |  |
| Gli influencer sui social media |  |  |  |  |  |
| I politici |  |  |  |  |  |
| I personaggi pubblici |  |  |  |  |  |
| I medici e i professionisti sanitari |  |  |  |  |  |
| Amici, conoscenti e colleghi |  |  |  |  |  |
| Familiari |  |  |  |  |  |

**La sua fiducia nelle istituzioni pubbliche cantonali è cambiata durante il COVID-19?**

- 1- La mia fiducia è diminuita
- 2- La mia fiducia è leggermente diminuita
- 3- La mia fiducia è rimasta la stessa
- 4- La mia fiducia è leggermente aumentata
- 5- La mia fiducia è aumentata

*<se risposta = 3 (costante) allora saltare domanda successiva>*

**Perché è cambiata?**

|  |
| --- |

**La sua fiducia nelle istituzioni pubbliche federali è cambiata durante il COVID-19?**

- 1- La mia fiducia è diminuita
- 2- La mia fiducia è leggermente diminuita
- 3- La mia fiducia è rimasta la stessa
- 4- La mia fiducia è leggermente aumentata
- 5- La mia fiducia è aumentata

*<se risposta = 3 (costante) allora saltare domanda successiva>*

**Perché è cambiata?**

|  |
| --- |

**La sua fiducia nella scienza è cambiata durante il COVID-19?**

- 1- La mia fiducia è diminuita
- 2- La mia fiducia è leggermente diminuita
- 3- La mia fiducia è rimasta la stessa
- 4- La mia fiducia è leggermente aumentata
- 5- La mia fiducia è aumentata

*<se risposta = 3 (costante) allora saltare domanda successiva>*

**Perché è cambiata?**

|  |
| --- |

**Credo che durante la pandemia le decisioni delle istituzioni pubbliche siano state guidate da…**

|  | 1- per niente | 2 | 3 | 4 | 5 – moltissimo |
| --- | --- | --- | --- | --- | --- |
| Interessi economici |  |  |  |  |  |
| Interessi politici |  |  |  |  |  |
| Interessi di salute pubblica |  |  |  |  |  |
| Interessi sociali |  |  |  |  |  |
| Altro:____________ |  |  |  |  |  |

**Le istituzioni (es. UFSP, Cantone) hanno emanato diverse restrizioni durante il periodo pandemico. Quanto si è trovato d’accordo con le diverse decisioni?**

|  | 1- per niente d’accordo | 2 | 3 | 4 | 5 – completamente d’accordo |
| --- | --- | --- | --- | --- | --- |
| Sono state chiuse le scuole, hanno limitato gli accessi alle case per anziani (Marzo 2020) |  |  |  |  |  |
| È stato inserito l’obbligo di mascherina nei trasporti pubblici e hanno limitato gli ingressi dai paesi a rischio (Luglio 2020) |  |  |  |  |  |
| Hanno reso disponibile l’app per il tracciamento SwissCovid (Agosto 2020) |  |  |  |  |  |
| Hanno raccomandato il telelavoro (Ottobre 2020) |  |  |  |  |  |
| Sono stati chiusi i ristoranti (Dicembre 2020) |  |  |  |  |  |
| Hanno limitato gli incontri tra persone a un numero massimo di 5, reso obbligatorio il telelavoro e chiuso i negozi di beni non necessari (Gennaio 2021) |  |  |  |  |  |
| È stata avviata la campagna vaccinale (Gennaio 2021) |  |  |  |  |  |
| Hanno allentato i provvedimenti riaprendo negozi, musei e incontri all’aperto (Febbraio 2021) |  |  |  |  |  |
| Hanno raccomandato il tampone ai primi sintomi (Marzo 2021) |  |  |  |  |  |
| Hanno nuovamente aperto ristoranti e bar all’aperto, le strutture per il tempo libero e quelle sportive e le lezioni in presenza all’università (Aprile 2021) |  |  |  |  |  |
| Nella fase di stabilizzazione hanno riaperto bar e ristoranti e allentato i limiti per gli incontri privati e le manifestazioni (Maggio 2021) |  |  |  |  |  |
| Hanno reso disponibile il Certificato Covid (Giugno 2021) |  |  |  |  |  |
| Hanno reso obbligatorio il Certificato Covid per i maggiori di 16 anni nei luoghi chiusi (Settembre 2021) |  |  |  |  |  |

**Il COVID-19 è una malattia grave**

- 1- per nulla d’accordo
- 2
- 3
- 4
- 5 – Completamente d’accordo

**Il COVID-19 è rischioso per la salute di molti**

- 1- per nulla d’accordo
- 2
- 3
- 4
- 5 – Completamente d’accordo

**È importante mettere in campo misure di prevenzione contro il COVID-19**

- 1 – per nulla d’accordo
- 2
- 3
- 4
- 5 – Completamente d’accordo

**Durante la pandemia abbiamo visto diverse posizioni su vari aspetti. In che misura è d’accordo con le seguenti affermazioni?**

|  | 1 – per niente d’accordo | 2 | 3 | 4 | 5 – completamente d’accordo |
| --- | --- | --- | --- | --- | --- |
| Le istituzioni sono completamente trasparenti |  |  |  |  |  |
| La comunità scientifica internazionale e la ricerca medica sono fondamentali per capire come gestire la pandemia |  |  |  |  |  |
| La salute è più importante dell’economia |  |  |  |  |  |
| I vaccini sono uno strumento importante per arginare la pandemia |  |  |  |  |  |
| È importante assicurare sempre che ogni cittadino sia libero di fare ciò che vuole |  |  |  |  |  |
| Il bene della comunità vale più della libertà del singolo |  |  |  |  |  |
| Il sistema sanitario sta trascurando i bisogni di altri pazienti non covid |  |  |  |  |  |
| Il governo ascolta e prende in considerazione i punti di vista dei cittadini |  |  |  |  |  |
| Il governo ascolta e prende in considerazione i punti di vista gli esperti |  |  |  |  |  |
| Nelle misure di prevenzione dal COVID-19 il governo considera equamente tutte le varie categorie professionali |  |  |  |  |  |
| È importante che le istituzioni decidano come la nazione debba comportarsi |  |  |  |  |  |
| La vaccinazione sta contribuendo a risolvere il problema del COVID-19 |  |  |  |  |  |
| Il Certificato Covid è importante per arginare la pandemia |  |  |  |  |  |
| Le istituzioni stanno facendo di tutto per risolvere il problema del COVID-19 |  |  |  |  |  |
| La Svizzera rispetto ad altre nazioni sta agendo bene per arginare la pandemia |  |  |  |  |  |
| Le varie restrizioni alla mobilità internazionale sono state adeguate (viaggi, frontalieri) |  |  |  |  |  |
| Le istituzioni hanno fatto troppo affidamento sulla responsabilità personale dei cittadini |  |  |  |  |  |

**Lei è:**

- - Vaccinato per il COVID-19
  - Non vaccinato per il COVID-19
  - Preferisco non rispondere
